# Obituary

**Published:** 1880-11

**Authors:** 


### Obituary.

### RESOLUTIONS IN MEMORY OF THEODORE B. CHURCH.

WHEREAS, It has pleased the Supreme Judge of the Universe to call from amongst us our esteemed friend and associate, the late Theodore B. Church, whose early decease was due to his untiring efforts in the exercise of professional services toward those placed under his charge; and

WHEREAS, We have been deprived of the companionship and counsel of our brother student, whose ability and learning, unsullied reputation and high sense of honor, have endeared him to the memory of his associates and made our mutual intercourse pleasant and agreeable; it is highly proper and right that we should show with what feelings of deep regret and sorrow we learned of his untimely death; Therefore

*Resolved*, That in the early decease of Theodore B. Church, the students of Rush Medical College have lost a useful, wise and good brother; the college a devoted and exemplary member, and the profession one of her most promising sons.

*Resolved*, That his generous, manly character and studious habits, command our admiration, and his example our earnest following.

*Resolved*, That to the family of the deceased we tender our heartfelt sympathy in this their time of bereavement and mourning.

*Resolved*, That his customary seat in the lecture-room be draped with the emblems of sorrow.

*Resolved*, That a copy of these resolutions be sent to the family of the deceased, and a copy also inserted in the daily papers of Chicago and Rockford, and the medical journals of this city.

C. G. SHIPMAN,<br>

C. E. WINSLOW,<br>

A. GARWOOD, } *Committee.*

RUSH MEDICAL COLLEGE, Sept. 29, 1880.

